# Biomarkers for the diagnosis and management of cardiovascular-kidney-metabolic syndrome: recent advances and prospects for clinical application

**DOI:** 10.3389/fendo.2026.1989794

**Published:** 2026-10-21

**Authors:** Liuqi Hu, Min Yang

**Affiliations:** 1 Changzhou Medical Center, Nanjing Medical University, Changzhou, China; 2 The First People’s Hospital of Changzhou, Changzhou, China

**Keywords:** biomarkers, cardiovascular-kidney-metabolic syndrome, chronic kidney disease, inflammation, insulin resistance, oxidative stress, risk stratification

## Abstract

Cardiovascular-kidney-metabolic (CKM) syndrome describes a multidirectional network linking dysfunctional adiposity, metabolic risk factors, kidney disease and cardiovascular injury. Its early stages are often clinically silent, while obesity, impaired kidney function, multimorbidity and recent exercise can alter the concentrations and interpretation of commonly used biomarkers. This narrative review, informed by a structured literature search, evaluates biomarkers across screening, risk stratification, treatment monitoring and precision phenotyping, while distinguishing CKM-specific evidence from evidence extrapolated from chronic kidney disease, diabetes, obesity and heart-failure populations. Established clinical biomarkers, including estimated glomerular filtration rate, urine albumin-to-creatinine ratio, B-type natriuretic peptide/N-terminal pro-B-type natriuretic peptide and high-sensitivity cardiac troponin, remain the foundation of clinical assessment. Emerging evidence spans directly measured markers of inflammation, oxidative stress and organ injury; indices derived from routine clinical variables; and metabolomic, lipidomic and other multi-omics platforms. Most of these measures provide biological or prognostic information but remain investigational because prospective external validation, analytical standardisation, actionable thresholds, incremental net benefit and biomarker-guided intervention evidence are limited. Lifestyle and exercise act both as upstream modifiers of CKM biology and as interpretive factors for several biomarkers. A clinically useful CKM biomarker framework should therefore integrate evidence maturity, population relevance, cross-organ specificity, serial trajectories, cost and equity. Emerging biomarkers should complement rather than replace established assessment until prospective studies demonstrate that their use changes treatment decisions and improves patient outcomes.

## 1 Introduction

In recent years, the prevalence of obesity, diabetes, chronic kidney disease (CKD) and cardiovascular disease (CVD) has continued to rise, posing a major global public-health challenge (1). In 2023, CVD accounted for 437 million disability-adjusted life years (DALYs) and 19.2 million deaths. Compared with 1990, DALYs increased from approximately 320 million to 437 million (approximately 1.37-fold), whereas deaths increased from 13.1 million to 19.2 million (approximately 1.47-fold) (2). The global prevalence of CKD has also risen from 378 million to 788 million (3). The prevalence of diabetes has doubled from approximately 7% to 14%, more than 1 billion people are living with obesity, and an estimated 1.54 billion people have metabolic syndrome (4–6). Although these conditions have traditionally been managed within separate disciplines, they share extensive pathophysiological interactions. The 2023 American Heart Association (AHA) Presidential Advisory therefore defined cardiovascular-kidney-metabolic (CKM) syndrome as a systemic disorder arising from interactions among metabolic risk factors, CKD and the cardiovascular system (7). CKM is best understood as a multidirectional network of reciprocal metabolic, kidney and cardiovascular interactions rather than a fixed linear sequence. Under the combined influence of dysfunctional adiposity, insulin resistance, inflammation, oxidative stress, vascular dysfunction and neurohormonal activation, clinically silent abnormalities may progress to overt multi-organ disease. The 2026 joint guideline from the AHA, American College of Cardiology (ACC), American Diabetes Association (ADA) and American Society of Nephrology (ASN) classifies CKM into five stages and emphasises the early identification of modifiable metabolic, kidney and cardiovascular risk factors (8). Nearly 90% of US adults meet the criteria for CKM stage 1 or higher, and preliminary longitudinal evidence, including conference data from the Atherosclerosis Risk in Communities (ARIC) cohort, suggests that some middle-aged adults transition to higher CKM stages over time (9, 10). Early screening, accurate risk assessment and coordinated management are therefore essential.

Biomarkers can support the detection and interpretation of biological processes that precede overt disease. Established clinical biomarkers, including estimated glomerular filtration rate (eGFR), urine albumin-to-creatinine ratio (UACR), B-type natriuretic peptide (BNP)/N-terminalpro-B-type natriuretic peptide (NT-proBNP) and high-sensitivity cardiac troponin (hs-cTn), remain central to clinical assessment. However, multimorbidity can distort their concentrations and meaning: obesity may lower natriuretic peptide concentrations, reduced kidney function may increase them, and sarcopenia may cause creatinine-based eGFR to overestimate kidney function (8, 11–15). Emerging evidence also includes directly measured molecular biomarkers, indices derived from routine clinical variables and multi-omics signatures. These analytically distinct categories should not be grouped together simply as novel biomarkers, because they differ in assay requirements, validation status and clinical actionability.

Existing reviews have generally catalogued biomarkers within individual diseases or organs, particularly CKD, diabetes, obesity or heart failure. Evidence generated in these populations cannot automatically be considered CKM-specific because CKM syndrome involves multidirectional interactions among dysfunctional adiposity, metabolic risk factors, kidney disease and cardiovascular injury. Previous reviews have also not consistently distinguished biological association from prognostic prediction, incremental predictive value, external validation and actionable clinical utility. The principal knowledge gap is therefore not a shortage of candidate biomarkers, but the absence of a clinically interpretable framework that links biomarkers to CKM network biology while defining the limits of the supporting evidence.

Accordingly, this review aims to: (1) map established and emerging biomarkers to the multidirectional pathophysiological network of CKM syndrome; (2) classify the evidence supporting each biomarker as biological association, prognostic prediction, incremental predictive value, external validation or demonstrated clinical utility; (3) distinguish CKM-specific studies from evidence extrapolated from CKD, diabetes, obesity, heart-failure or other component populations; and (4) identify the analytical, clinical, economic and equity requirements that must be met before emerging biomarkers can be incorporated into routine CKM management.

## 2 Literature search and evidence appraisal

This article is a narrative review informed by a structured literature search. PubMed was searched from database inception through 24 September 2026. No additional bibliographic databases were searched. The search combined terms for cardiovascular-kidney-metabolic syndrome and CKM health with terms related to biomarkers, screening, diagnosis, prognosis, risk assessment, treatment monitoring, precision medicine, phenotyping, proteomics, metabolomics, lipidomics and multi-omics. Because contemporary CKM terminology is relatively recent, supplementary searches also combined chronic kidney disease, diabetes, obesity, heart failure, cardiovascular disease, metabolic syndrome and cardiorenal syndrome with domain-specific terms related to adiposity, insulin resistance, inflammation, oxidative stress, derived indices, cardiac and kidney injury, fibrosis, inter-organ communication, omics, exercise, lifestyle medicine and clinical utility.

Search results were screened by title and abstract, followed by full-text assessment when necessary. Evidence selection prioritised clinical guidelines and consensus statements, systematic reviews and meta-analyses, prospective cohort studies, externally validated prediction studies, randomised controlled trials and biomarker-guided intervention studies. Cross-sectional studies, retrospective cohorts, secondary analyses of large databases and mechanistic studies were included when they informed biological association, hypothesis generation or exploratory risk prediction. Duplicate reports and studies without a relevant biomarker–phenotype or biomarker–outcome relationship were not used to support the principal clinical conclusions. Purely animal or experimental studies were used selectively to explain biological mechanisms and were not treated as evidence of clinical utility. Conference abstracts were not used to support the principal clinical conclusions; when no full-length publication was available, a conference abstract was considered only as preliminary or hypothesis-generating evidence and was explicitly identified as such.

Evidence maturity was classified as follows: Tier 1, established clinical use supported by standardised assays and guideline-based roles; Tier 2, replicated prognostic or adjunctive value without evidence for independent treatment decisions; Tier 3, reported incremental value but limited or absent independent external validation; and Tier 4, biological association or early research-stage evidence. Biological association, prognostic prediction, incremental predictive value, external validation and demonstrated clinical utility were treated as distinct levels of evidence. Tier assignment is specific to the proposed clinical use and the evidence population. Replication requires distinct participant cohorts rather than repeated analyses of the same source dataset. Tier 2 does not establish CKM-specific external validation or a treatment-guiding threshold. These tiers are descriptive categories for this narrative review, not formal Grading of Recommendations Assessment, Development and Evaluation (GRADE) ratings.

Evidence was considered CKM-specific only when the study population was explicitly defined or staged using contemporary CKM criteria. Evidence from chronic kidney disease, diabetes, obesity, heart-failure, cardiorenal or general cardiovascular populations was classified as extrapolated component-population evidence. Because this is a narrative review rather than a systematic review or meta-analysis, study selection was purposive and evidence-informed rather than exhaustive; no formal risk-of-bias instrument, GRADE assessment or quantitative evidence synthesis was applied.

## 3 Biomarker-relevant network pathophysiology of CKM syndrome

CKM syndrome should be viewed as a dynamic system of reciprocal interactions among adipose tissue, metabolic pathways, the kidneys, the vasculature and the heart, rather than as a one-way progression from metabolic dysfunction to end-organ disease. Excess and dysfunctional adiposity, insulin resistance, kidney dysfunction, vascular injury, neurohormonal activation, inflammation, oxidative stress and cardiac remodelling interact through reciprocal feedback loops. Consequently, biomarkers should not be interpreted as pathway-specific or organ-specific in isolation: a change in a single marker may reflect several simultaneous processes, including altered production, clearance, tissue injury, haemodynamic stress, recent exercise and treatment effects. This section therefore focuses on mechanisms that directly influence biomarker selection, concentration or clinical interpretation.

### 3.1 Core mechanisms

CKM syndrome arises from a multidirectional network rather than a linear sequence of injury. Five closely interconnected mechanisms—excess and dysfunctional adiposity, insulin resistance, chronic low-grade inflammation, oxidative stress, and fibrosis and tissue remodelling—jointly link metabolic dysfunction with kidney and cardiovascular injury. These mechanisms frequently coexist and reinforce one another; biomarkers reflecting them should therefore be interpreted as overlapping biological signals rather than as indicators of isolated pathways or organs (7, 16).

#### 3.1.1 Excess and dysfunctional adiposity

Excess adiposity and adipose-tissue dysfunction are important upstream drivers of CKM syndrome. The American Heart Association defines CKM stage 1 by excess adiposity, dysfunctional adipose tissue or both, highlighting the roles of visceral and ectopic fat in linking metabolic, kidney and cardiovascular abnormalities (7, 8). When chronic energy excess exceeds the safe storage capacity of subcutaneous adipose tissue, adipocyte hypertrophy, local hypoxia, fibrosis, macrophage infiltration, dysregulated lipolysis and adipokine imbalance develop. Increased free-fatty-acid flux and ectopic fat deposition in the liver, skeletal muscle, pancreas, epicardial and perirenal regions promote insulin resistance, lipotoxicity, inflammation, oxidative stress, endothelial dysfunction and neurohormonal activation, thereby contributing to hypertension, type 2 diabetes mellitus (T2DM), metabolic dysfunction-associated steatotic liver disease, albuminuria, CKD and CVD (7, 16). Adiposity-related biomarkers should therefore be interpreted according to fat burden, distribution and function rather than body weight alone.

#### 3.1.2 Insulin resistance

Insulin resistance (IR) links dysfunctional adiposity and type 2 diabetes mellitus with glomerular hyperfiltration, endothelial dysfunction and impaired myocardial energy metabolism. Hyperglycaemia, lipotoxicity and adipokine imbalance promote kidney and cardiac injury through interconnected pathways involving advanced glycation end-product/receptor signalling, renin-angiotensin-aldosterone system activation, oxidative stress and impaired phosphoinositide 3-kinase/protein kinase B/endothelial nitric oxide synthase signalling. These abnormalities reduce nitric oxide bioavailability and promote microvascular injury, kidney fibrosis and cardiac remodelling (16–23). IR-related biomarkers primarily reflect systemic metabolic stress and should therefore be interpreted alongside adiposity, glycaemic status and evidence of organ injury.

#### 3.1.3 Chronic low-grade inflammation

Chronic low-grade inflammation connects dysfunctional adipose tissue and insulin resistance with progressive cardiovascular and kidney injury. Adipocyte hypertrophy, tissue hypoxia and macrophage infiltration increase circulating inflammatory mediators, including C-reactive protein, interleukin-6 and tumour necrosis factor-alpha, thereby aggravating insulin resistance, endothelial dysfunction and tissue fibrosis. In CKD, uraemic toxins, gut microbiota dysbiosis and volume overload further amplify systemic and intrarenal inflammation, contributing to tubular injury, myocardial damage and heart failure (16, 24–39). Inflammatory biomarkers are not CKM-specific and should be interpreted in relation to adiposity, kidney function, acute illness and other causes of systemic inflammation.

#### 3.1.4 Oxidative stress

Oxidative stress results from an imbalance between reactive oxygen species production and antioxidant defences and amplifies inflammation and tissue remodelling. Hyperglycaemia, lipotoxicity, uraemic toxins, mitochondrial dysfunction and nicotinamide adenine dinucleotide phosphate oxidase activation increase oxidative burden, damaging podocytes, tubular epithelial cells, vascular endothelium and cardiomyocytes. These processes contribute to albuminuria, kidney fibrosis, myocardial hypertrophy and vascular remodelling (16, 24, 33, 40–46). However, oxidative-stress biomarkers are sensitive to specimen handling, assay methods and short-term biological variation; the lack of analytical standardisation currently limits their use mainly to mechanistic and research settings.

#### 3.1.5 Fibrosis and tissue remodelling

Fibrosis and tissue remodelling represent common downstream responses to persistent metabolic, inflammatory, oxidative and haemodynamic stress. Activation of transforming growth factor-beta/Smad signalling promotes fibroblast activation and extracellular-matrix deposition, resulting in myocardial stiffness and adverse remodelling as well as tubular atrophy, interstitial fibrosis and nephron loss. Fibrotic burden may also accumulate in other metabolically affected organs, including the liver (47–66). Because fibrosis-related biomarkers may originate from several organs and reflect shared remodelling pathways, they should be interpreted as indicators of multisystem structural injury rather than as markers of a single organ or causal mechanism.

Overall, these five mechanisms form reciprocal feedback loops rather than a strict pathological hierarchy. Excess adiposity and adipose-tissue dysfunction promote insulin resistance, inflammation and oxidative stress, whereas kidney and cardiovascular dysfunction can reduce physical capacity, alter metabolism and further aggravate adipose-tissue dysfunction. This network-based interpretation provides the biological basis for combining biomarkers across metabolic, inflammatory, kidney and cardiovascular domains. These interconnected mechanisms and the potential modifying influences of exercise and lifestyle are illustrated in **Figure 1**.

**Figure 1:**
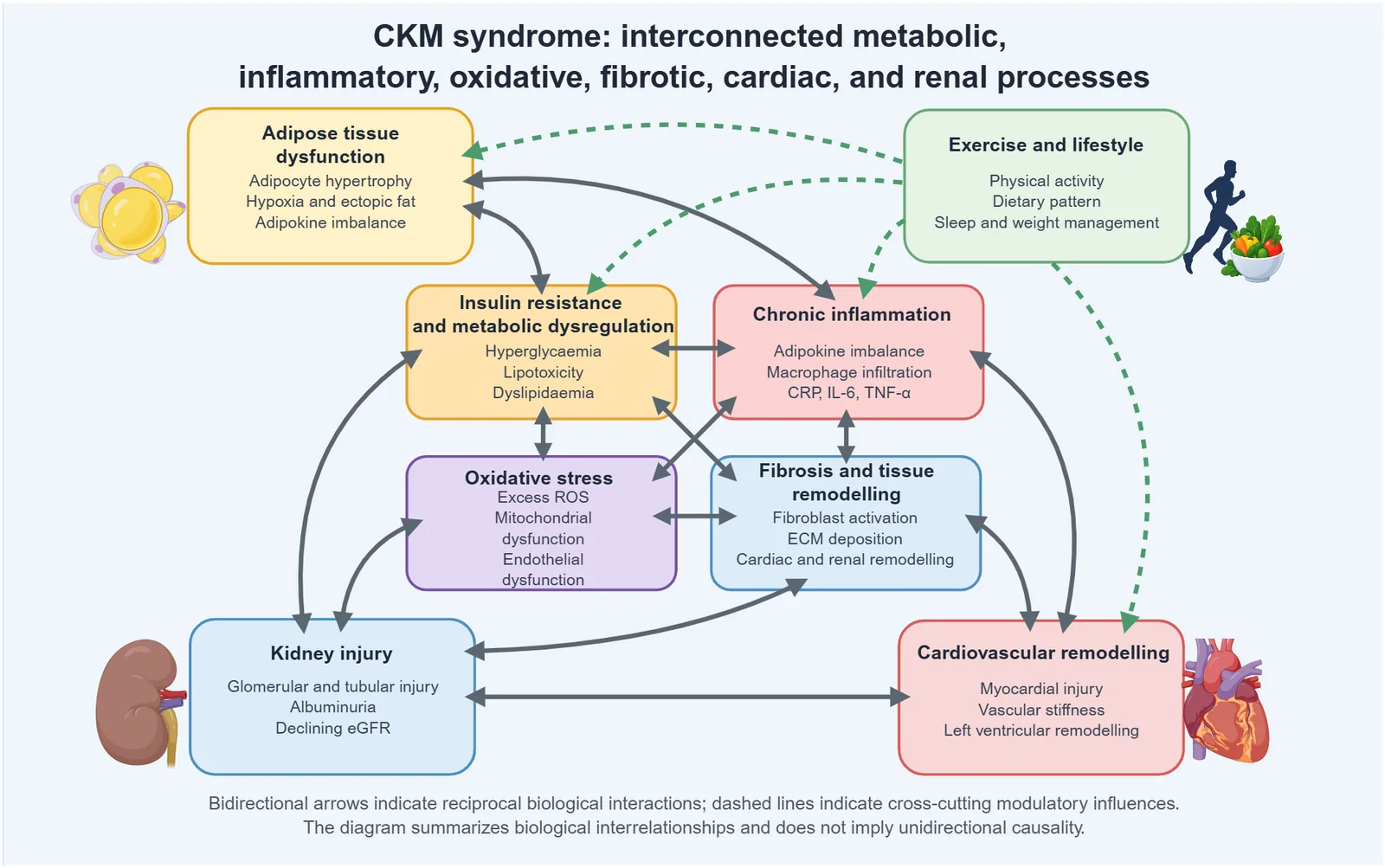
Interconnected mechanisms and organ injury in cardiovascular-kidney-metabolic (CKM) syndrome. Adipose-tissue dysfunction, insulin resistance and metabolic dysregulation, chronic inflammation, oxidative stress, and fibrosis and tissue remodelling form an interacting network linked to kidney injury and cardiovascular remodelling. Adipose-tissue dysfunction is a core mechanism, with reciprocal links to metabolic dysregulation and inflammation. Solid double-headed arrows indicate representative reciprocal biological interactions, including kidney injury with metabolic dysregulation, oxidative stress, fibrosis and cardiovascular remodelling, and cardiovascular remodelling with inflammation and fibrosis. Connections are selectively displayed for readability rather than as an exhaustive map: inflammation also contributes to kidney and tubular injury, and kidney dysfunction can amplify inflammation, although this reciprocal connection is not separately drawn. Green dashed arrows indicate potential modulatory influences of exercise and lifestyle on adipose-tissue dysfunction, insulin resistance, inflammation and cardiovascular remodelling. The diagram summarises biological relationships and does not establish causality or imply a fixed sequence. CRP, C-reactive protein; ECM, extracellular matrix; eGFR, estimated glomerular filtration rate; IL-6, interleukin-6; ROS, reactive oxygen species; TNF-α, tumour necrosis factor-α.

### 3.2 Lifestyle and exercise as biological and interpretive modifiers

Lifestyle behaviours and exercise should be considered across all CKM stages because they influence both disease mechanisms and biomarker interpretation. Energy balance, dietary quality, physical activity, sleep, smoking, psychosocial stress and the social environment modulate adipose-tissue distribution, skeletal-muscle glucose uptake, mitochondrial function, insulin sensitivity, endothelial function, blood pressure, hepatic fat accumulation, inflammation and autonomic regulation. Regular aerobic and resistance exercise can improve cardiorespiratory fitness, insulin sensitivity, blood pressure, lipid profiles and vascular function even when weight loss is modest (67, 68). For individuals with overweight or obesity, exercise should form part of a comprehensive weight-management programme incorporating dietary energy restriction and behavioural support (67). Exercise prescriptions should be tailored to functional capacity, cardiovascular status and comorbidities, particularly in patients with CKD, heart failure, sarcopenia, frailty or limited exercise tolerance (13, 68).

Lifestyle interventions also influence longitudinal biomarker profiles. In individuals with obesity, sustained weight loss can reduce visceral and hepatic fat and improve insulin sensitivity and selected metabolic measures (69). Lifestyle interventions may also lower high-sensitivity C-reactive protein and leptin concentrations, while adiponectin responses vary across populations and intervention types (70, 71). These findings derive largely from studies in obesity, diabetes and other selected populations; their magnitude and consistency across CKM stages remain incompletely established. Assessment of treatment response should therefore extend beyond body mass index (BMI) to include weight trajectory, waist circumference, physical fitness and relevant metabolic measures, with blood pressure, glycaemia, lipids, UACR, eGFR and liver measures monitored according to the individual’s clinical profile.

Acute exercise responses should be distinguished from adaptations to sustained training. Recent prolonged or high-intensity exercise can transiently increase hs-cTn, serum creatinine, creatine kinase, urinary albumin and selected inflammatory markers; dehydration and haemoconcentration may further influence measured concentrations (72–74). For longitudinal comparisons, sampling time, hydration status and recent exercise exposure should be documented and standardised where feasible, with fasting conditions matched when relevant to the assay. For planned UACR sampling or confirmation, strenuous exercise should be avoided for at least 24 h beforehand because exercise within the preceding 24 h can transiently elevate UACR independently of kidney damage (75). Persistent albuminuria should be confirmed by repeat testing after transient causes have been considered, preferably using a first-morning urine sample (13, 75). After prolonged or high-intensity exercise, hs-cTn commonly peaks approximately 2–6 h after exercise cessation and returns towards baseline within 24–72 h; the time course varies with the exercise exposure and individual clinical context (72). This expected time course can inform interpretation of serial results, but elevated hs-cTn should not be attributed to exercise alone or used to defer assessment of possible acute myocardial injury. Symptoms, electrocardiographic findings, serial measurements and other clinical findings remain essential (72). Obesity may lower BNP and NT-proBNP concentrations despite clinically relevant cardiac stress, whereas lipoprotein(a) is predominantly genetically determined and generally changes little with lifestyle intervention (76). Biomarker trajectories should therefore be interpreted in light of each marker’s biological determinants and expected response, rather than used as a uniform measure of lifestyle-intervention success.

### 3.3 Mechanism-biomarker mapping

In a network model of CKM syndrome, biomarkers map to overlapping processes rather than to a fixed sequence of isolated mechanisms. A single marker may reflect changes in production, release, distribution, clearance and treatment response across several organs. Growth differentiation factor 15 (GDF-15), for example, reflects cellular stress and inflammation from multiple tissue sources; neutrophil gelatinase-associated lipocalin (NGAL) may indicate tubular injury but can also increase with systemic inflammation; and natriuretic peptides integrate myocardial stress, haemodynamic load, adiposity and kidney clearance. Mechanism-based classification is therefore useful for organising evidence, but it should not be interpreted as proof of organ specificity or causality. Clinical assessment should combine complementary biomarkers with the patient’s phenotype, serial trajectories and relevant confounders. Representative mechanistic, component-disease and cross-organ syntheses further support this network interpretation (77–86).

## 4 Biomarker classification and research progress

For clarity, we distinguish three analytically and clinically different categories. Established clinical biomarkers, including serum creatinine-based eGFR, UACR, BNP/NT-proBNP and hs-cTn, have standardised assays and established roles in diagnosis, staging, risk assessment or safety monitoring. Emerging directly measured biomarkers, including kidney injury molecule-1 (KIM-1), NGAL, soluble tumour necrosis factor receptors 1 and 2 (sTNFR1/2), soluble urokinase-type plasminogen activator receptor (suPAR), soluble suppression of tumorigenicity 2 (sST2), galectin-3 and GDF-15, generally require additional assays and remain predominantly adjunctive or investigational. Derived composite indices, including triglyceride-glucose index (TyG), estimated glucose disposal rate (eGDR), neutrophil-to-lymphocyte ratio (NLR), systemic immune-inflammation index (SII), systemic inflammation response index (SIRI), albumin-to-neutrophil/lymphocyte ratio (ANLR), albumin-to-globulin ratio (AGR) and neutrophil-to-high-density lipoprotein cholesterol ratio (NHR), are calculated from existing clinical or laboratory variables and do not necessarily require new analytical infrastructure; however, their formulae, thresholds, validation and clinical utility remain heterogeneous. This classification reflects analytical requirements and clinical maturity rather than biological novelty alone.

Evidence maturity is reported consistently using the four-tier framework in Section 2 and **Table 1**: Tier 1, established clinical use supported by standardised assays and guideline-based roles; Tier 2, replicated prognostic or adjunctive value without evidence for independent treatment decisions; Tier 3, reported incremental value but limited or absent independent external validation; and Tier 4, biological association or early research-stage evidence.

**Table 1:** Evidence matrix for established and emerging CKM biomarkers and assessment tools.

Biomarker/domain	Core claim/clinical use	Evidence population	Data source	Study design	Evidence tier	External validation	CKM specificity	Cross-cutting	Clinical maturity	Key limitation	References
Kidney function and damage: eGFR; UACR	Guideline-supported CKD diagnosis/staging, risk assessment, treatment eligibility and safety monitoring.	CKD, diabetes, CVD and contemporary CKM populations	Guidelines, clinical cohorts and therapeutic trials	Guideline synthesis; prospective cohorts; RCTs	Tier 1	Extensive multi-cohort validation	Established across component conditions; incorporated into CKM staging	Yes	Established	Creatinine is affected by muscle mass and diet; UACR varies with infection, glycaemia, blood pressure, hydration and recent strenuous exercise. Confirm persistence.	(8, 13, 14, 74, 75, 87–94)
Cardiac stress and injury: BNP/NT-proBNP; hs-cTn	Supports heart-failure evaluation and prognostic refinement and may prompt imaging or specialist assessment when clinically indicated.	Heart failure, CKD, diabetes and general cardiovascular cohorts	Guidelines, clinical cohorts and randomised trials	Prospective cohorts; guideline synthesis; RCTs	Tier 1	Extensive for defined cardiovascular uses	Mostly extrapolated component-population evidence	Yes	Established for defined indications	Affected by adiposity, kidney function, age, rhythm and chronic injury; hs-cTn may rise transiently after prolonged/high-intensity exercise. Use clinical context and serial change.	(8, 72, 95–102)
Adjunctive filtration: cystatin C; eGFRcr-cys	Refines GFR estimation when creatinine is unreliable, including altered muscle mass.	CKD and general populations	Diagnostic-accuracy and outcome cohorts	Diagnostic accuracy; prospective outcomes	Tier 2	Externally validated for GFR refinement	Extrapolated component-population evidence	No (filtration-focused)	Validated adjunct	Influenced by inflammation, thyroid status, corticosteroids and adiposity; not CKM-specific.	(14, 88, 148)
Direct inflammatory markers: CRP/hs-CRP, cytokines, sTNFR1/2, suPAR	Provides biological and prognostic information but does not independently direct CKM therapy.	Predominantly CKD, diabetes, obesity and CVD cohorts	Clinical cohorts, reviews and mechanistic studies	Prospective/retrospective observational; mechanistic	Tier 2–4	Variable; stronger for prognosis than actionability	Mostly extrapolated; limited prospective CKM-defined cohorts	Yes	Adjunctive/investigational	Not CKM-specific; affected by acute infection, adiposity, autoimmune disease, medication and kidney clearance. Few actionable thresholds or guided trials.	(103–107)
Direct oxidative-stress markers: MDA, F_2_-isoprostanes, AOPP, 8-OHdG; antioxidant measures	Supports mechanistic and exploratory phenotyping rather than routine clinical decisions.	Diabetes, CKD, atherosclerosis and selected CVD cohorts	Small clinical cohorts and experimental studies	Cross-sectional; mechanistic; small cohorts	Tier 4	Limited independent validation	Extrapolated component-population evidence	Yes	Investigational	Specimen handling, short-term variability and platform heterogeneity limit comparability; harmonised reference intervals and thresholds are lacking.	(108–112)
Derived inflammatory, dietary and redox indices: NLR, SII, SIRI, ANLR, AGR, NHR, DII/E-DII, OBS, CDAI	Accessible surrogate indicators for population-level association and exploratory risk refinement.	General and CKM-staged populations	NHANES, CHARLS and other large secondary datasets	Cross-sectional; secondary observational analyses	Tier 3–4	Limited; reused source cohorts are not independent replication	Some CKM-specific analyses; substantial extrapolation	Yes	Investigational	Sensitive to infection, nutrition, medication and organ function; heterogeneous formulae/cut-offs; incremental value, calibration, net benefit and treatment thresholds are generally unproven.	(122–133)
Derived insulin-resistance, adiposity and lipid indices: eGDR, TyG derivatives, CVAI, VAI, LAP, AIP, NHHR, RC	May refine metabolic/adiposity phenotyping and risk estimates, but should not independently determine treatment.	CKM stages 0–3, older adult and general populations	UK Biobank, GOLD-Health and Chinese/general cohorts	Large prospective observational; secondary analyses	Tier 3	Limited independent geographic and clinical validation	Partly CKM-specific	Yes	Investigational/exploratory adjunct	Population-specific thresholds and reproducible incremental value beyond established variables and PREVENT, decision-curve benefit and treatment utility remain insufficient.	(113–121, 134, 135, 170–175)
Adipose-tissue dysfunction: leptin, adiponectin, adiponectin-to-leptin ratio, FABP4	Complements mechanistic assessment of adipose dysfunction and exploratory risk refinement.	Obesity, metabolic syndrome and CKD cohorts	Component-disease cohorts, including a prospective FABP4 study	Mechanistic; cross-sectional; selected prospective cohorts	Tier 3–4	FABP4 prospective evidence exists; CKM-specific validation limited	Mostly extrapolated component-population evidence	Yes	Investigational	Affected by sex, body composition, inflammation and kidney function; adiponectin may rise in advanced CKD and should not automatically imply greater protection.	(170, 176–179)
Cardiac remodelling, fibrosis and congestion: sST2, galectin-3, GDF-15, H-FABP, CA125, bio-ADM, MR-proADM and FGF-23	May support adjunctive assessment of myocardial stress, fibrosis, tissue congestion, endothelial-barrier dysfunction and haemodynamic stress in selected settings, but should not determine treatment intensity in isolation.	Predominantly heart-failure, CKD and CVD cohorts	Prospective cohorts, trial substudies and mechanistic studies	Prospective observational; trial substudy; mechanistic	Tier 2–4	Marker-dependent; prognostic replication exceeds treatment-guidance evidence	Mostly extrapolated component-population evidence	Yes	Adjunctive/investigational	Signals are not organ-specific; assay availability, thresholds and evidence that marker-guided management improves outcomes remain limited.	(136–139, 141–147, 183, 184, 197, 220)
Kidney tubular injury and cellular stress: KIM-1, NGAL, L-FABP, NAG, urinary IL-18, [TIMP-2]×[IGFBP7]	May identify acute tubular injury or stress in defined settings; monitoring utility in stable CKM is unproven.	AKI, CKD, diabetes and critical-care cohorts	Diagnostic/prognostic cohorts and mechanistic studies	Diagnostic accuracy; prognosis; mechanistic	Tier 2–4	Variable; strongest in defined AKI settings	Predominantly extrapolated; limited stable-CKM evidence	Yes	Adjunctive in selected acute settings/otherwise investigational	AKI performance cannot be directly extrapolated to chronic CKM; inflammation, assay platform and setting affect specificity; treatment thresholds are absent.	(149–153, 180)
Metabolomics and lipidomics	Supports exploratory molecular subtyping and candidate-panel development, not routine CKM decisions.	Diabetes, CKD, CVD and selected CKM populations	Discovery cohorts and small validation datasets	Cross-sectional discovery; nested case-control; small validation	Tier 4	Limited independent replication and platform harmonisation	Mostly extrapolated; CKM-specific evidence remains early	Yes	Investigational	Diet, medication, microbiome, kidney clearance and analytical platform influence signals; parsimony, calibration, cost and clinical net benefit require prospective evaluation.	(154–161)
Other omics and computational platforms: proteomics, transcriptomics, epigenomics, ncRNAs, EVs, integrated multi-omics and AI/ML	Enables hypothesis generation, inter-organ communication research and exploratory phenotyping.	Discovery datasets and selected population cohorts	High-dimensional molecular and model-development datasets	Discovery; model development; mechanistic	Tier 4	Generally insufficient independent external validation	Limited CKM-specific prospective evidence	Yes	Investigational	At risk of overfitting, batch effects and data leakage; translation requires reproducibility, calibration, equity assessment, explicit action rules and biomarker-guided outcome benefit.	(81, 154, 155, 162–167, 169, 175, 221–223)
Lifestyle and recent exercise (interpretive modifier)	Chronic lifestyle change can alter CKM biology, whereas recent strenuous exercise can transiently alter hs-cTn, creatinine and UACR.	Obesity, diabetes, exercise-physiology and CKD populations	Consensus statements, guidelines, intervention studies and acute-exercise cohorts	Consensus/guideline; intervention; acute physiology	Tier 2–4	Variable by exposure and biomarker	Extrapolated component-population evidence	Yes	Established interpretive context; not a stand-alone biomarker	Exercise dose, timing, hydration and sampling are heterogeneous; elevated hs-cTn must not be attributed to exercise without clinical assessment.	(13, 67–75)

8-OHdG, 8-Hydroxy-2′-deoxyguanosine; AGR, albumin-to-globulin ratio; AI, artificial intelligence; AIP, atherogenic index of plasma; AKI, acute kidney injury; ANLR, albumin-to-neutrophil/lymphocyte ratio; AOPP, advanced oxidation protein products; BNP, B-type natriuretic peptide; CA125, carbohydrate antigen 125; CHARLS, China Health and Retirement Longitudinal Study; CKD, chronic kidney disease; CKM, cardiovascular-kidney-metabolic syndrome; CRP, C-reactive protein; CVAI, chinese visceral adiposity index; CVD, cardiovascular disease; DII, Dietary Inflammatory Index; E-DII, Energy-adjusted Dietary Inflammatory Index; EV, extracellular vesicle; FABP4, fatty acid-binding protein 4; GDF-15, growth differentiation factor 15; GFR, glomerular filtration rate; H-FABP, heart-type fatty acid-binding protein; IGFBP7, insulin-like growth factor-binding protein 7; IL, interleukin; KIM-1, kidney injury molecule-1; L-FABP, liver-type fatty acid-binding protein; LAP, lipid accumulation product; MDA, malondialdehyde; MR-proADM, mid-regional pro-adrenomedullin; NAG, N-acetyl-β-D-glucosaminidase; NGAL, neutrophil gelatinase-associated lipocalin; NHHR, non-high-density lipoprotein cholesterol-to-high-density lipoprotein cholesterol ratio; NHR, neutrophil-to-high-density lipoprotein cholesterol ratio; NLR, neutrophil-to-lymphocyte ratio; NT-proBNP, N-terminal pro-B-type natriuretic peptide; OBS, Oxidative Balance Score; PREVENT, Predicting Risk of Cardiovascular Disease EVENTs; SII, systemic immune-inflammation index; SIRI, systemic inflammation response index; TIMP-2, tissue inhibitor of metalloproteinases 2; TyG, triglyceride-glucose index; UACR, urine albumin-to-creatinine ratio; VAI, visceral adiposity index; bio-ADM, biologically active adrenomedullin; eGDR, estimated glucose disposal rate; eGFR, estimated glomerular filtration rate; eGFRcr-cys, estimated glomerular filtration rate based on creatinine and cystatin C; hs-CRP, high-sensitivity C-reactive protein; hs-cTn, high-sensitivity cardiac troponin; ncRNA, non-coding ribonucleic acid; sST2, soluble suppression of tumorigenicity 2; sTNFR1/2, soluble tumour necrosis factor receptors 1 and 2.

Tier 1, established clinical use supported by standardised assays and guideline-based roles; Tier 2, replicated prognostic or adjunctive value without evidence for independent treatment decisions; Tier 3, reported incremental value but limited or absent independent external validation; and Tier 4, biological association or early research-stage evidence. Categories are non-exclusive because many markers are cross-cutting. CKM-specific evidence requires explicit contemporary CKM definition or staging; evidence from CKD, diabetes, obesity, heart failure or general CVD populations is labelled as extrapolated component-population evidence. Repeated analyses of overlapping NHANES, UK Biobank, CHARLS or other source cohorts do not constitute independent external replication.

### 4.1 Established clinical biomarkers

Traditional biomarkers remain a crucial foundation for the screening, diagnosis, staging and risk assessment of CKM syndrome. Serum creatinine and its derivative, eGFR, are core indicators for evaluating renal function; a low eGFR is closely associated with the risk of renal function deterioration, cardiovascular events and mortality (8, 13, 87). However, creatinine is influenced by non-renal factors such as muscle mass, age, sex, diet and medication, and may lead to misinterpretation of renal function in patients with sarcopenia, older adults or those with obesity; furthermore, its sensitivity to early-stage renal damage is limited, and different calculation formulas also affect the comparability of results (14, 88–91). The UACR reflects damage to the glomerular barrier and vascular endothelium and is associated with the progression of CKD and cardiovascular risk; however, it is influenced by factors such as blood pressure, blood glucose, infection and physical activity, and exhibits significant intra-individual variability. Recent vigorous or prolonged exercise can also produce transient albuminuria. An elevated UACR should therefore be confirmed under clinically stable conditions after reversible factors—including recent strenuous exercise, infection, marked hyperglycaemia and uncontrolled blood pressure—have been considered (13, 74, 75). Some patients may have low eGFR but no albuminuria; therefore, the use of UACR alone may fail to detect atypical renal damage (13, 75, 87, 92–94).

BNP and NT-proBNP reflect myocardial wall stress and volume load and aid in heart-failure screening and prognostic stratification; however, reduced renal function can lead to decreased clearance and consequently elevated levels. Age and atrial fibrillation also interfere with interpretation, while levels may be lower in obese individuals. The applicability of a uniform cut-off value is limited in the CKM population, and a comprehensive assessment combining renal function, body fat and heart rhythm is required (8, 95–98).High-sensitivity cardiac troponin I (hs-cTnI) and high-sensitivity cardiac troponin T (hs-cTnT) can sensitively identify myocardial damage and predict adverse outcomes; however, in patients with CKD or cardiorenal syndrome, levels are often persistently elevated due to factors such as ongoing subclinical damage and left ventricular hypertrophy. Because chronic hs-cTn elevation lacks specificity for acute myocardial infarction, interpretation must rely on symptoms, electrocardiographic findings and serial changes (99–102). Recent prolonged or high-intensity exercise is another important interpretive factor because it may cause a transient rise in hs-cTn. The timing and intensity of recent exercise should therefore be documented, and post-exercise elevations should be interpreted together with symptoms, electrocardiographic findings, baseline values and serial changes (72).

Established biomarkers have standardised assays, broad availability and guideline-supported roles, but they predominantly reflect organ function, stress or injury rather than the full CKM network. They should be complemented by clinical phenotype, imaging and serial assessment rather than replaced by a single emerging marker.

Although these biomarkers are clinically actionable, much of their supporting evidence was generated in CKD, diabetes, heart-failure or general cardiovascular populations rather than in cohorts prospectively defined by CKM criteria. Their established roles therefore arise from standardised assays, guideline-supported indications and demonstrated clinical utility across CKM component conditions, not from CKM-specific validation alone.

### 4.2 Emerging biomarkers, derived indices and omics platforms

Emerging CKM markers differ substantially in their analytical requirements, biological meaning and clinical maturity. To avoid conflating newly measured molecular analytes with indices calculated from routine clinical data, this section separates the evidence into four categories: directly measured inflammatory and oxidative-stress biomarkers; derived inflammatory, metabolic and lipid indices; biomarkers of cardiac and kidney injury and inter-organ cross-talk; and metabolomic, lipidomic and other omics platforms. Within each category, biological association is distinguished from prognostic prediction, incremental predictive value, external validation and demonstrated clinical utility. Evidence derived from CKD, diabetes, obesity or heart-failure populations is also distinguished from evidence generated in cohorts defined specifically by CKM criteria. Inflammatory and oxidative-stress markers and related indices are summarised in **Figure 2**.

**Figure 2:**
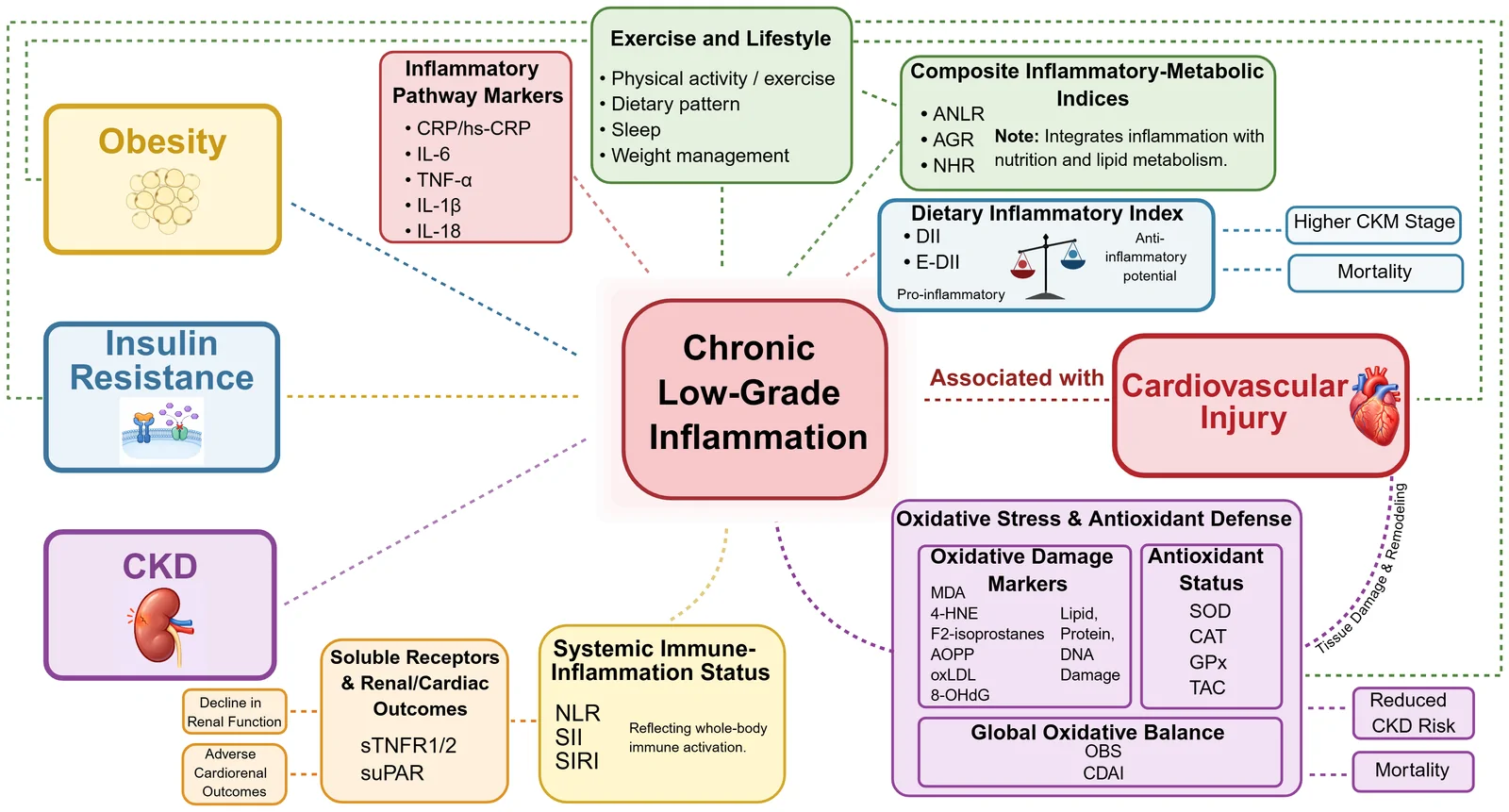
Inflammatory and oxidative-stress markers and indices in CKM syndrome. The schematic places obesity, insulin resistance and chronic kidney disease alongside chronic low-grade inflammation, measured pathway markers, derived indices, oxidative-stress measures and clinical outcomes. Exercise and lifestyle are shown as potential modifiers of adiposity, insulin resistance, inflammatory, oxidative and cardiovascular pathways. The green dashed outlines and connections include the obesity and insulin-resistance domains and indicate these cross-cutting influences; they do not represent a validated intervention pathway. Outcome labels summarise observational associations reported for particular indices and populations rather than effects of biomarker-guided treatment. In particular, lower CKD risk and mortality associations concern separate OBS/CDAI analyses and should not be interpreted as demonstrated benefits of antioxidant therapy or uniform findings for every marker in the panel. Dashed connections summarise reported associations or potential modifying relationships and do not establish causality. CKM, cardiovascular-kidney-metabolic; CKD, chronic kidney disease; CRP, C-reactive protein; hs-CRP, high-sensitivity C-reactive protein; IL, interleukin; TNF-α, tumour necrosis factor-α; ANLR, albumin-to-neutrophil/lymphocyte ratio; AGR, albumin-to-globulin ratio; NHR, neutrophil-to-high-density lipoprotein cholesterol ratio; DII, Dietary Inflammatory Index; E-DII, Energy-adjusted Dietary Inflammatory Index; NLR, neutrophil-to-lymphocyte ratio; SII, systemic immune-inflammation index; SIRI, systemic inflammation response index; sTNFR1/2, soluble tumour necrosis factor receptors 1 and 2; suPAR, soluble urokinase-type plasminogen activator receptor; MDA, malondialdehyde; 4-HNE, 4-Hydroxynonenal; AOPP, advanced oxidation protein products; oxLDL, oxidised low-density lipoprotein; 8-OHdG, 8-Hydroxy-2′-deoxyguanosine; SOD, superoxide dismutase; CAT, catalase; GPx, glutathione peroxidase; TAC, total antioxidant capacity; OBS, Oxidative Balance Score; CDAI, Composite Dietary Antioxidant Index; DNA, deoxyribonucleic acid.

#### 4.2.1 Directly measured biomarkers of inflammation and oxidative stress

Directly measured inflammatory biomarkers include C-reactive protein (CRP), high-sensitivity CRP (hs-CRP), interleukin-6, tumour necrosis factor-alpha, interleukin-1beta, interleukin-18, soluble tumour necrosis factor receptors 1 and 2 (sTNFR1/2), and soluble urokinase-type plasminogen activator receptor (suPAR). They reflect overlapping components of innate immune and acute-phase activation, but are not specific to CKM syndrome and may be influenced by acute infection, adiposity, autoimmune disease, medication use and kidney function. sTNFR1/2 and suPAR have been associated with kidney-function decline and adverse cardiovascular or kidney outcomes, although most supporting evidence derives from CKD, diabetes or cardiovascular cohorts rather than prospectively defined CKM populations (103–107).

Oxidative-stress measures can be grouped into products of lipid, protein or deoxyribonucleic acid (DNA) oxidation and indicators of antioxidant defence. Representative measures include F2-isoprostanes, malondialdehyde, advanced oxidation protein products, 8-hydroxy-2’-deoxyguanosine, antioxidant enzymes and total antioxidant capacity (108–111). Although abnormal concentrations have been associated with diabetes, CKD, atherosclerosis and cardiovascular remodelling, these assays remain sensitive to sample collection, storage, analytical platform and short-term biological variation. The lack of harmonised methods, reference intervals and actionable thresholds limits their current use mainly to mechanistic or exploratory research. Moreover, associations with oxidative-stress markers do not establish that antioxidant supplementation or nonspecific antioxidant treatment improves clinical outcomes; therapeutic translation remains limited by target specificity, dose, timing and the physiological roles of redox signalling (112).

Overall, directly measured inflammatory and oxidative-stress biomarkers provide biologically relevant information, but an association with CKM stage or prognosis does not establish clinical actionability. Except for routinely available markers such as CRP or hs-CRP in selected clinical contexts, most lack CKM-specific external validation and evidence that biomarker-guided intervention improves patient outcomes.

#### 4.2.2 Derived inflammatory, metabolic and lipid indices

Derived indices combine routinely available haematological, biochemical, dietary or anthropometric variables to approximate inflammation, nutritional status, oxidative balance, insulin resistance, adiposity or atherogenic lipid burden. Representative measures include NLR, SII, SIRI, ANLR, AGR, NHR, Dietary Inflammatory Index (DII), energy-adjusted DII (E-DII), Oxidative Balance Score (OBS), Composite Dietary Antioxidant Index (CDAI), eGDR, TyG and its anthropometric derivatives, atherogenic index of plasma (AIP), non-high-density lipoprotein cholesterol-to-high-density lipoprotein cholesterol ratio (NHHR) and remnant cholesterol (113–121). The relevant evidence should be attributed to specific indices and outcomes. In CKM-defined observational analyses, SII and SIRI were associated with all-cause and cardiovascular mortality (122); ANLR with these mortality outcomes in advanced CKM (123); AGR with all-cause and cardiovascular mortality in CKM (124); and NHR with CKM status and cardiovascular mortality (125). DII and E-DII estimate the inflammatory potential of dietary intake (126, 127), with CKM studies reporting associations with CKM risk or severity and mortality (128, 129).

Higher OBS was associated with CKM stage and mortality risk in an NHANES analysis (130), whereas a separate cross-sectional NHANES study associated higher CDAI with lower odds of advanced CKM (131). In a UK Biobank analysis, E-DII and CDAI were associated with incident CKD across CKM stages (132). Higher OBS was also associated with lower incident CKD risk in a general adult cohort, which constitutes component-population evidence rather than validation in a contemporary CKM-defined cohort (133).

TyG has been evaluated as a surrogate for insulin resistance in a systematic review of diagnostic accuracy, rather than as a CKM treatment-selection test (134). In a prospective CHARLS cohort with CKM stages 0-3, eGDR was associated with incident CVD and was evaluated alongside other insulin-resistance surrogates (135). Their accessibility does not, however, establish that they improve decisions beyond established clinical variables and validated risk models.

These findings should be interpreted cautiously. Many reports are cross-sectional studies or secondary analyses of overlapping National Health and Nutrition Examination Survey (NHANES), UK Biobank, China Health and Retirement Longitudinal Study (CHARLS) and other large datasets; separate publications therefore cannot automatically be regarded as independent replication. Formula definitions, measurement units, covariate adjustment and data-derived cut-offs vary across studies. Most indices lack prospective external validation and have not shown reproducible improvements in discrimination, calibration, net reclassification, integrated discrimination or decision-curve net benefit beyond tools such as Predicting Risk of Cardiovascular Disease EVENTs (PREVENT). No validated thresholds or biomarker-guided trials support treatment selection or dose adjustment based on these indices alone. The evidence should therefore be viewed as hypothesis-generating or adjunctive rather than independently actionable.

#### 4.2.3 Biomarkers of cardiac and kidney injury and inter-organ cross-talk

CKM syndrome is characterised by continuous bidirectional interactions among metabolic, kidney and cardiovascular systems. Biomarkers of organ injury and cross-organ signalling may therefore complement conventional measures, but their concentrations frequently reflect multiple biological processes and should not be assigned to a single organ or pathway without qualification.

Emerging cardiovascular biomarkers include soluble suppression of tumorigenicity 2 (sST2), galectin-3, growth differentiation factor 15 (GDF-15) and heart-type fatty acid-binding protein (H-FABP). sST2 reflects mechanical stress, inflammation and pro-fibrotic signalling, whereas galectin-3 is associated with macrophage activation, fibrosis and adverse remodelling. GDF-15 has multiple tissue sources and reflects cellular stress, inflammation and overall disease burden; H-FABP is released following cardiomyocyte injury and may provide supplementary prognostic information in heart failure (136–140). However, most evidence derives from heart-failure or cardiovascular cohorts, and these markers lack CKM-specific treatment thresholds validated in randomised trials. Circulating collagen-turnover products are likewise promising fibrosis-related adjuncts but remain insufficiently validated for routine CKM decisions (141).

Carbohydrate antigen 125 (CA125), biologically active adrenomedullin (bio-ADM) and mid-regional pro-adrenomedullin (MR-proADM) provide information related to fluid retention, tissue congestion, endothelial-barrier dysfunction and haemodynamic stress. They may support phenotypic assessment but should not independently determine treatment intensity. Fibroblast growth factor 23 (FGF-23), which increases in CKD and is associated with mineral-metabolism abnormalities, left-ventricular hypertrophy and mortality, illustrates kidney-to-heart signalling but is not a cardiac-specific biomarker (142–147).

Kidney-related biomarkers extend assessment beyond glomerular filtration to tubular injury, inflammation and cellular stress. Cystatin C is less dependent on muscle mass than serum creatinine, and the combined creatinine-cystatin C equation can improve glomerular filtration rate (GFR) estimation when creatinine is unreliable. Cystatin C should nevertheless be regarded as an adjunctive filtration marker rather than a CKM-specific biomarker (148). Kidney injury molecule-1 (KIM-1), neutrophil gelatinase-associated lipocalin (NGAL), liver-type fatty acid-binding protein (L-FABP), N-acetyl-beta-D-glucosaminidase (NAG) and urinary interleukin-18 reflect different aspects of tubular epithelial injury or intrarenal inflammation (149–151). Their concentrations may rise before serum creatinine in acute injury, but evidence for routine monitoring of stable CKM remains limited. The urinary product of tissue inhibitor of metalloproteinases 2 and insulin-like growth factor-binding protein 7 ([TIMP-2] ×[IGFBP7]) has an established role in short-term acute kidney injury risk assessment in critically ill adults and should not be extrapolated directly to chronic CKM management. Prospective and review evidence for urinary tubular-injury markers has largely arisen from diabetes and CKD populations rather than contemporary CKM-defined cohorts (152, 153).

Thus, cystatin C has a defined adjunctive role in GFR assessment, while most other cardiac and kidney injury markers remain prognostic or mechanistic candidates. Clinical adoption requires CKM-specific validation, standardised assays, evidence of incremental value beyond conventional markers and demonstration that biomarker-guided decisions improve outcomes.

#### 4.2.4 Metabolomics, lipidomics and other omics platforms

Metabolomics and lipidomics provide system-level information on insulin resistance, substrate utilisation, mitochondrial dysfunction, lipotoxicity and interactions between host metabolism, gut microbiota and kidney clearance. Candidate signatures include amino acids, acylcarnitines, glycerophospholipids, sphingolipids, ceramides and glycerides. Gut-derived or host-microbial co-metabolites, including trimethylamine N-oxide, N,N,N-trimethyl-5-aminovaleric acid, indoxyl sulphate and p-cresyl sulphate, may reflect combined effects of dietary exposure, microbial metabolism and kidney clearance (154–161). These measures may support exploratory CKM subtyping, but their concentrations are strongly influenced by diet, medication use, microbiome composition, kidney function and analytical methods.

Non-coding ribonucleic acids (RNAs) and extracellular vesicles are potential mediators and biomarkers of cross-organ communication, while integrated proteomic, metabolomic, lipidomic, transcriptomic, genomic and spatial data may help identify molecular CKM subtypes (162–167). Artificial-intelligence and machine-learning approaches can integrate these data with clinical variables, imaging and longitudinal digital measures, but performance should be assessed using external validation, calibration, interpretability, clinical net benefit and subgroup equity rather than discrimination alone (168). Extracellular-vesicle and non-coding-RNA evidence remains predominantly mechanistic or discovery-phase (169).

These platforms remain predominantly in discovery or early validation phases. Before clinical implementation, they require harmonised sample collection and analytical procedures, replication in independent CKM populations, parsimonious model development, comparison with established risk tools, assessment of cost and feasibility, and prospective evidence that their use improves clinical decisions or patient outcomes. **Figure 3** summarises inter-organ communication, multi-omics integration and proposed pathways to clinical translation.

**Figure 3:**
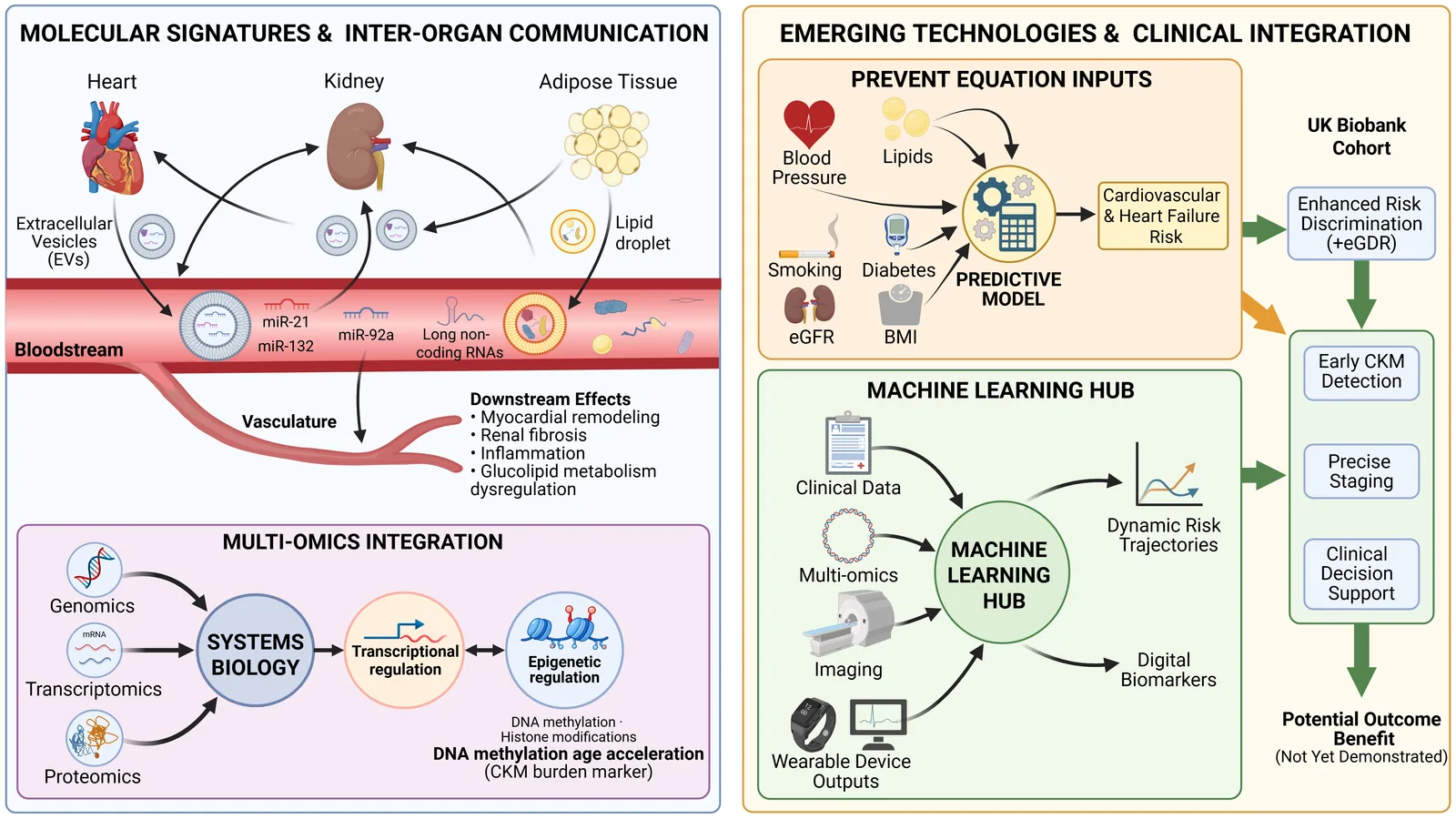
Inter-organ communication, multi-omics integration, and potential clinical translation in cardiovascular–kidney–metabolic (CKM) syndrome. The left panel depicts bidirectional signalling among adipose tissue, the heart, and kidneys through the bloodstream and vasculature, involving extracellular vesicles, non-coding RNAs, and lipid-related signals. These processes are associated with myocardial remodelling, renal fibrosis, inflammation, and dysregulated glucolipid metabolism. Genomic, transcriptomic and proteomic data feed into systems-biology integration, which connects to transcriptional and epigenetic regulation. Epigenetic regulation, including DNA methylation and histone modifications, interacts bidirectionally with transcriptional regulation; DNA methylation age acceleration is shown as a marker of CKM burden. The right-panel labels describe proposed applications rather than established clinical benefits of an integrated platform. The terminal label, “Potential outcome benefit (not yet demonstrated)”, explicitly indicates that improved patient outcomes have not been demonstrated for the depicted combined workflow. The right panel illustrates PREVENT equation inputs, cohort-based assessment of estimated glucose disposal rate (eGDR), and machine-learning integration of clinical, multi-omics, imaging, and wearable-device data as potential approaches to risk discrimination, dynamic assessment, early detection, staging, and clinical decision support. Routine adoption requires analytical reproducibility, independent external validation, calibration, equitable subgroup performance, incremental clinical benefit, feasible action rules, and evidence that biomarker-guided care improves outcomes. EVs, extracellular vesicles; miR, microRNA; RNA, ribonucleic acid; mRNA, messenger RNA; DNA, deoxyribonucleic acid; PREVENT, Predicting Risk of Cardiovascular Disease EVENTs; eGFR, estimated glomerular filtration rate; BMI, body mass index.

### 4.3 Adiposity-related measures and biomarkers of adipose-tissue dysfunction

Assessment of adiposity in cardiovascular-kidney-metabolic (CKM) syndrome should consider fat burden, fat distribution, and biological function. Body mass index (BMI), interpreted together with waist circumference, remains the most accessible approach to identifying general and abdominal obesity, although BMI cannot distinguish fat mass from lean mass or characterise visceral and ectopic fat distribution (7, 8). Computed tomography and magnetic resonance imaging can quantify visceral and ectopic fat depots, including hepatic and epicardial fat, more directly; however, cost, limited accessibility, and, in the case of computed tomography, exposure to ionising radiation constrain their routine use in population screening (170).

Several derived indices integrate anthropometric and metabolic information. The Chinese visceral adiposity index (CVAI), visceral adiposity index (VAI), and lipid accumulation product (LAP) were developed as surrogate measures of visceral adiposity, adipose-tissue dysfunction, or lipid overaccumulation (171–173), whereas triglyceride-glucose (TyG)-related anthropometric indices, such as TyG-waist circumference (TyG-WC), combine a surrogate measure of insulin resistance with information on body size or fat distribution (116, 174). In prospective observational cohorts of individuals with CKM stages 0–3, higher CVAI and TyG-WC values were associated with incident cardiovascular disease (116, 174). Although one UK Biobank analysis reported improvements in the area under the receiver-operating-characteristic curve, net reclassification improvement, and integrated discrimination improvement after TyG-related indices were added to a base prediction model, reproducible incremental value beyond established clinical variables or externally validated models such as PREVENT, together with population-specific thresholds, independent external validation, and clinical net benefit, remains insufficiently established (116, 175). Accordingly, these indices should currently be regarded as tools for research and exploratory risk refinement rather than as stand-alone determinants of clinical decisions.

At the circulating molecular level, leptin, adiponectin, and the adiponectin-to-leptin ratio may provide complementary information on adipose-tissue dysfunction (176, 177). Obesity-associated hyperleptinaemia and reduced adiponectin concentrations generally reflect an adverse metabolic phenotype, whereas the adiponectin-to-leptin ratio may better represent the balance between pro-inflammatory leptin signalling and the insulin-sensitising and anti-inflammatory actions of adiponectin (176, 177). Circulating fatty acid-binding protein 4 (FABP4) independently predicted incident metabolic syndrome during five years of follow-up in a prospective cohort; however, its incremental clinical utility and external validation in CKM-specific populations remain limited (178).

These circulating markers are not specific to CKM syndrome. Their concentrations may vary according to sex, body composition, metabolic status, systemic inflammation, and kidney function (176, 177, 179). In advanced chronic kidney disease, circulating adiponectin concentrations may be paradoxically elevated and have been associated with adverse outcomes; therefore, a higher adiponectin concentration should not automatically be interpreted as indicating greater metabolic protection (179). At present, adipokines and FABP4 are most appropriately used in mechanistic research and exploratory risk stratification. They should not replace routine clinical assessment based on BMI, waist circumference, conventional metabolic risk factors, estimated glomerular filtration rate, urine albumin-to-creatinine ratio, and validated cardiovascular-risk tools (7, 8, 175).

## 5 Clinical applications of biomarkers

Established biomarkers, emerging directly measured biomarkers, derived indices and omics platforms differ in terms of the maturity of evidence supporting their use in CKM management. eGFR, UACR, BNP/NT-proBNP and hs-cTn are already used in clinical screening, staging or risk assessment; most emerging biomarkers, derived indices and omics platforms primarily provide supplementary insights into mechanisms or prognosis. The following discussion outlines their applicability across four scenarios: screening, risk prediction, treatment monitoring and precision stratification (**Table 2**).

**Table 2:** Evidence-aligned integration of CKM biomarkers into comprehensive management.

Clinical task	Tier 1 foundation	Adjunctive/investigational options	Use in practice	Safeguards and references
Kidney screening	Serum creatinine/eGFR plus UACR	Cystatin C/eGFRcr-cys when creatinine is unreliable; tubular markers mainly for defined or research settings	Identify CKD, stratify risk and confirm abnormalities persisting ≥3 months	Repeat after reversible causes, including strenuous exercise, infection, hyperglycaemia and uncontrolled blood pressure (8, 13, 14, 74, 75, 87–94, 148)
Cardiac screening	BNP/NT-proBNP and hs-cTn when clinically indicated	sST2, galectin-3, GDF-15, CA125 and bio-ADM in selected/research settings	Identify myocardial stress, injury or congestion and trigger imaging or specialist assessment	Interpret with symptoms, serial change, adiposity, kidney function, rhythm and recent exercise (8, 72, 95–102, 136–140, 142–147, 181–184, 197)
Metabolic/adiposity assessment	BMI, waist circumference, blood pressure, glucose/HbA1c and lipid profile	CVAI, VAI, LAP, TyG derivatives, adipokines and FABP4	Characterise modifiable risk and guide evidence-based lifestyle/weight management	Derived indices and adipokines do not independently determine therapy; thresholds and external validation remain limited (7, 8, 67–71, 113–121, 170–174, 176–179)
Primary-prevention risk	Validated clinical models such as PREVENT plus established organ markers	Derived inflammatory/metabolic indices and multi-marker panels	Estimate absolute risk and inform preventive intensity	Require calibration, incremental value and net benefit beyond the base model; avoid treating repeated analyses of one cohort as replication (106, 107, 112–133, 175, 185, 212)
CKD cardiorenal prognosis	eGFR, UACR and established cardiovascular markers	KIM-1, sTNFR1/2 and selected panels	Refine prognosis in defined CKD populations	CRIC evidence is internally validated in CKD rather than a universal CKM tool; incremental gains may be modest (87, 92, 107, 149, 152, 180, 185, 217)
Organ-specific treatment	eGFR, UACR, serum potassium, HbA1c, blood pressure and clinical phenotype	NT-proBNP/hs-cTn to prompt further assessment; congestion markers as adjuncts	Select and monitor therapies according to established CKD, diabetes, obesity or heart-failure indications	Biomarkers support indications and safety; they should not replace guideline-defined phenotype or determine dose alone (8, 13, 187–196, 201–204, 212)
Exploratory mechanistic phenotyping	Clinical phenotype plus Tier 1 biomarkers	hs-CRP, IL-6, sST2, galectin-3, GDF-15 and omics signatures	Generate testable hypotheses about inflammatory, fibrotic, metabolic or congestion-dominant states	A plausible drug-biomarker relationship is not actionable without reproducible interaction, threshold and outcome benefit (103–107, 136–138, 140, 155–167, 187, 197–200)
Temporal monitoring	Serial eGFR, UACR, creatinine, potassium, symptoms and congestion status	NT-proBNP, bio-ADM and other markers in selected contexts	Distinguish expected treatment responses from progressive injury and plan follow-up	A short-term biomarker improvement is not a substitute for outcome benefit; GUIDE-IT cautions against treating a biomarker target in isolation (187, 201–204, 215)
Precision phenotyping	Integrated clinical, laboratory and imaging phenotype	Tier 2–4 biomarkers, metabolomics, lipidomics, ncRNAs, EVs and AI models	Research stratification and development of parsimonious panels	Requires independent validation, calibration, interpretability, cost/equity assessment and prospective clinical-utility testing (16, 136–138, 140, 154–167, 175, 187, 197, 205–207, 212, 223)
Implementation and equity	Accessible routine tests in primary care	Conditional specialist or centralised testing for complex phenotypes	Match testing intensity to whether the result can change referral, treatment or monitoring	Report completion, misclassification, downstream cost, out-of-pocket burden and subgroup benefit; lack of high-cost testing must not delay indicated care (7, 216–219, 224)

AI, artificial intelligence; CA125, carbohydrate antigen 125; CKD, chronic kidney disease; CKM, cardiovascular-kidney-metabolic syndrome; CRP, C-reactive protein; EV, extracellular vesicle; GDF-15, growth differentiation factor 15; HbA1c, glycated haemoglobin; IL, interleukin; LAP, lipid accumulation product; NT-proBNP, N-terminal pro-B-type natriuretic peptide; PREVENT, Predicting Risk of Cardiovascular Disease EVENTs; TyG, triglyceride-glucose index; UACR, urine albumin-to-creatinine ratio; VAI, visceral adiposity index; bio-ADM, biologically active adrenomedullin; eGFR, estimated glomerular filtration rate; eGFRcr-cys, estimated glomerular filtration rate based on creatinine and cystatin C; hs-cTn, high-sensitivity cardiac troponin; ncRNA, non-coding ribonucleic acid; sST2, soluble suppression of tumorigenicity 2; sTNFR1/2, soluble tumour necrosis factor receptors 1 and 2.

### 5.1 Screening and early diagnosis

The early stages of CKM are typically characterised by an absence of overt clinical symptoms; however, metabolic risk factors, renal damage and subclinical cardiovascular abnormalities may persist for a long time prior to the occurrence of overt cardiovascular events (1, 8). Therefore, screening should be strengthened in high-risk populations, such as those with T2DM, obesity, metabolic syndrome and hypertension, to detect modifiable risk factors and subclinical organ damage at the earliest possible stage. Renal screening should be based on eGFR derived from serum creatinine and UACR. eGFR primarily reflects renal filtration function, while UACR reflects damage to the glomerular barrier and may indicate systemic microvascular risk. Combined assessment of these two parameters aids in the identification of renal dysfunction, with or without albuminuria (8, 13, 87). The diagnosis of CKD requires abnormalities in renal structure or function to persist for at least three months; therefore, after the initial detection of reduced eGFR or elevated UACR, the finding should be confirmed by repeat testing after previous results have been considered and acute or reversible causes excluded, including recent strenuous exercise, urinary tract infection, fever, marked hyperglycaemia, uncontrolled hypertension and acute haemodynamic instability (13, 74, 75). Where eGFR and UACR are inconsistent with clinical risk, or where early tubular damage is still suspected, KIM-1, NGAL, L-FABP and sTNFR1/2 may be measured in specific clinical or research settings to provide a supplementary assessment of tubular injury and inflammatory activation (13, 149, 180). With regard to the heart, for individuals at high risk of heart failure but without obvious symptoms, initial screening with BNP or NT-proBNP may be considered; where necessary, this may be combined with hs-cTn to identify subclinical myocardial damage, and further echocardiography and specialist assessment should be undertaken if test results are abnormal (8, 181, 182). Novel cardiovascular biomarkers may provide adjunctive information in selected clinical or research settings. For example, sST2, galectin-3, H-FABP, CA125, bio-ADM and FGF-23 may provide complementary information on myocardial inflammation and injury, fluid retention, tissue congestion and cardiorenal interactions (142, 147, 183, 184). As BMI, renal function and cardiac rhythm can all influence their concentrations, clinical interpretation should account for serial changes in these biomarkers within the overall clinical context.

### 5.2 Risk stratification and prognostic prediction

CKM risk assessment should focus on the dynamic progression of the disease from a healthy state, through single-disease conditions, to the coexistence of multiple conditions and ultimately death. Combined models can integrate complementary information from NT-proBNP, hs-cTn, eGFR, UACR, KIM-1 and sTNFR1/2; however, new indicators only have practical value if they consistently improve discriminatory power, calibration, risk reclassification and clinical net benefit compared to traditional models. The Chronic Renal Insufficiency Cohort (CRIC) four-variable model, comprising NT-proBNP, hs-cTnT, albuminuria and eGFR, was internally validated in 3,718 patients with CKD and is suitable for supplementary assessment of cardiovascular and renal outcome risks in individuals with confirmed CKD (185). The PREVENT equations, on the other hand, are designed for primary prevention in adults without clinical CVD; they are sex-specific, exclude race as a variable and can predict the risks of overall CVD, atherosclerotic cardiovascular disease (ASCVD) and heart failure. The addition of UACR, glycated haemoglobin (HbA1c) and the social deprivation index yields only a modest gain, and the model still requires recalibration in Asian and other healthcare systems (175). Consequently, the PREVENT framework is generally the preferred choice for individuals with CKM stages 0–3 who do not have clinical CVD, while in patients with established CKD, the CRIC model may provide complementary estimation of composite cardiovascular and kidney risk. Markers such as KIM-1 and sTNFR1/2 offer limited additional discriminatory power in existing models and are currently more suitable as supplementary tools in research settings (107). Multi-state models incorporating sleep and psychological factors can expand research into disease trajectories; however, their observational findings cannot yet be directly applied to causal decision-making (186).

### 5.3 Treatment guidance and efficacy monitoring

The clinical value of a biomarker lies not only in its association with risk but also in whether measurement changes treatment selection, safety monitoring or follow-up in a way that improves outcomes. A recently proposed three-dimensional framework may help operationalise biomarker-guided pharmacotherapy in CKM syndrome by integrating organ-specific, pathway-specific and temporal dimensions (187).

The organ-specific dimension links biomarkers of metabolic, kidney and cardiovascular dysfunction to evidence-based treatment indications and safety requirements. eGFR, UACR and serum potassium inform the initiation and monitoring of renin-angiotensin system inhibitors, sodium-glucose cotransporter-2 inhibitors and mineralocorticoid receptor antagonists. Guidelines and randomised trials support cardiorenal benefits when these therapies are prescribed according to established indications (8, 13, 188–196). Natriuretic peptides and hs-cTn refine cardiac risk and may trigger imaging or specialist assessment, but they should not independently determine drug choice or dose.

The pathway-specific dimension considers cross-system signals of inflammation, fibrosis, metabolic stress and congestion. hs-CRP, interleukin-6, galectin-3, GDF-15 and sST2 may support exploratory mechanistic phenotyping and research into drug-responsive biological states (136–138, 140, 197). However, biological plausibility does not establish clinical actionability. Treatment selection requires a reproducible marker-treatment interaction, an actionable threshold and evidence that marker-guided care improves outcomes. Studies of sodium-glucose cotransporter-2 inhibitors and semaglutide have reported changes in inflammatory markers (198, 199), whereas the canakinumab trial demonstrated the therapeutic relevance of a selected inflammatory pathway in patients with established atherosclerotic disease (200). These distinct findings do not currently justify routine CKM drug selection based solely on inflammatory-marker concentrations.

The temporal dimension evaluates serial biomarker trajectories as pharmacodynamic and safety signals. Expected treatment-related changes, such as the early eGFR dip after sodium-glucose cotransporter-2 inhibitor initiation, should be distinguished from progressive organ injury. Changes in UACR, eGFR, serum creatinine, serum potassium, natriuretic peptides and congestion-related markers should be interpreted with symptoms, haemodynamic status and long-term outcomes. Short-term biomarker improvement cannot substitute for clinical benefit, and Guiding Evidence-Based Therapy Using Biomarker Intensified Treatment in Heart Failure (GUIDE-IT) illustrates that intensifying therapy to achieve an NT-proBNP target is not necessarily superior to high-quality guideline-directed care (201–204).

This framework does not support treatment selection solely on the basis of investigational biomarkers. Biomarker-guided prescribing in CKM remains anchored in established clinical variables and biomarkers with validated treatment or safety implications. TyG, eGDR, KIM-1, sST2, galectin-3 and GDF-15 lack randomised-trial-validated treatment thresholds and should be viewed as tools for exploratory mechanistic phenotyping, risk refinement or research until prospective intervention studies show that their use improves decisions and outcomes.

### 5.4 Precision subtyping and individualised management

The current CKM staging system reflects overall disease burden, but dominant mechanisms and intervention priorities may differ among patients within the same stage (1, 8). A research framework combining mechanism-informed phenotypes with intervenability could distinguish metabolic-dominant, kidney-injury or inflammation-dominant, myocardial-stress or congestion-dominant, and fibrosis or remodelling-dominant patterns using complementary clinical and biomarker information (16, 154, 205). Treatment should nevertheless remain anchored in clinical phenotype and evidence-based indications. For example, GLP-1 receptor agonists or sodium-glucose cotransporter-2 inhibitors may be considered when supported by metabolic, cardiovascular or kidney indications; renin-angiotensin system inhibitors, sodium-glucose cotransporter-2 inhibitors and mineralocorticoid receptor antagonists should be selected and monitored using established measures such as eGFR, UACR and serum potassium; and heart-failure therapy should follow the relevant phenotype rather than an isolated NT-proBNP target (8, 13). TyG, eGDR, KIM-1, sST2, galectin-3, GDF-15, adipokines and omics signatures may support research phenotyping, but they are not independently validated criteria for drug selection (136–138, 140, 154, 196, 197, 201). Biomarker interpretation should also account for sex, age, adiposity and kidney function (175, 206, 207). This phenotype framework therefore remains investigational until its reproducibility, treatment interactions and capacity to improve outcomes are demonstrated.

CKM management should be viewed as an iterative network linking screening and early diagnosis, risk stratification, precision phenotyping, and treatment guidance and monitoring. Information generated at any point may prompt reassessment at another: treatment response can refine risk, risk assessment can trigger additional screening, and emerging phenotypic information can change follow-up intensity. Established biomarkers anchor this cycle, while research-stage markers should enter only when they address a defined clinical question and add information beyond validated models. Feasibility, cost and health equity modify every decision point (**Figure 4**).

**Figure 4:**
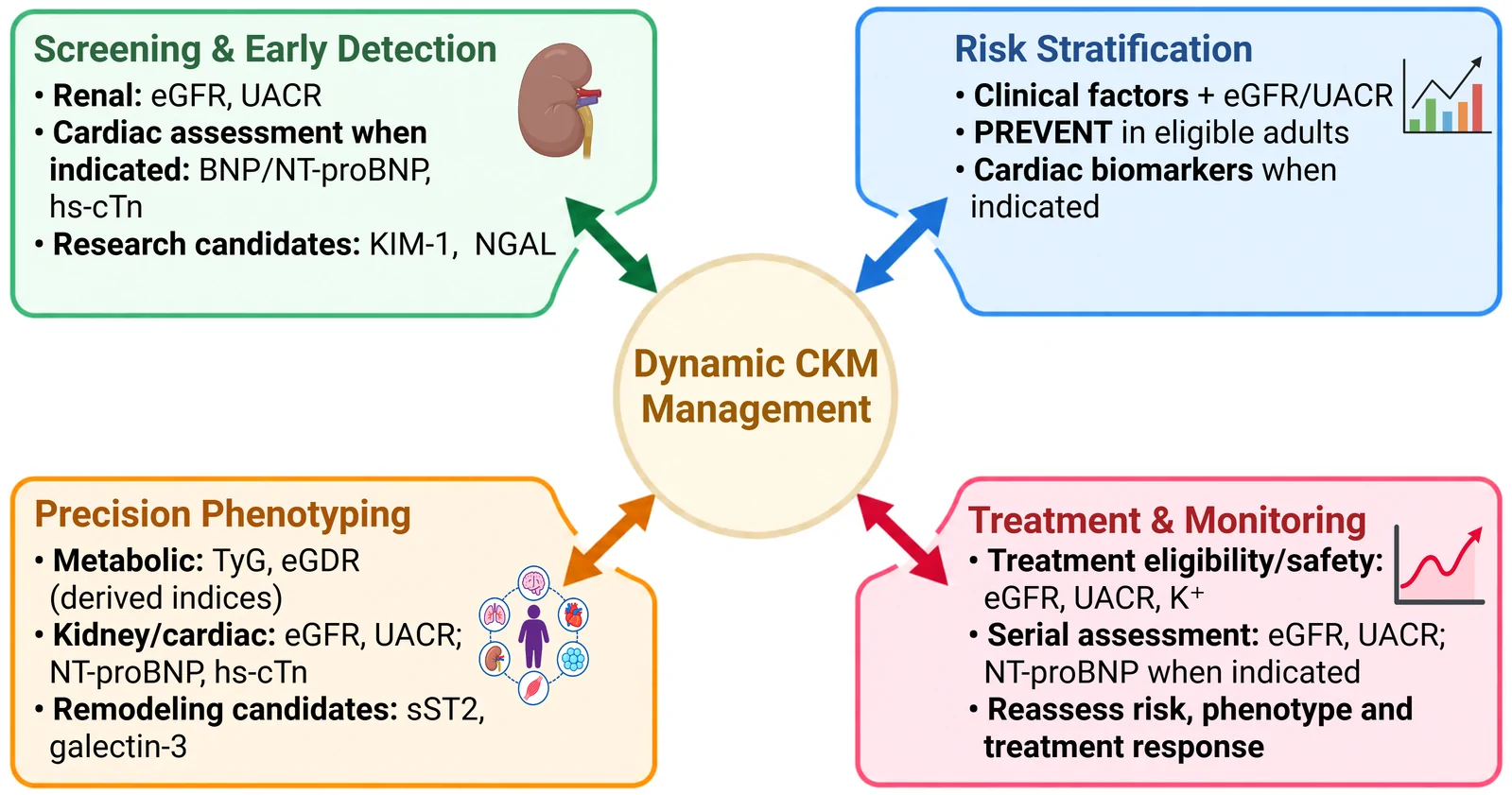
Dynamic biomarker-informed management of cardiovascular–kidney–metabolic (CKM) syndrome. Screening and early detection, risk stratification, precision phenotyping, and treatment monitoring are connected bidirectionally to a central management hub. The arrows represent iterative reassessment as clinical status, biomarker measurements, and treatment response change; they do not imply a fixed sequence. Established clinical measures are distinguished from routinely derived indices and research-stage candidate biomarkers. Cardiac biomarkers are assessed when clinically indicated, and research-stage candidates do not have validated thresholds for guiding CKM treatment. eGFR, estimated glomerular filtration rate; UACR, urine albumin-to-creatinine ratio; BNP, B-type natriuretic peptide; NT-proBNP, N-terminal pro-B-type natriuretic peptide; hs-cTn, high-sensitivity cardiac troponin; KIM-1, kidney injury molecule-1; NGAL, neutrophil gelatinase-associated lipocalin; TyG, triglyceride–glucose index; eGDR, estimated glucose disposal rate; sST2, soluble suppression of tumorigenicity 2; K^+^, potassium. Galectin-3 is a candidate marker of remodelling rather than an established treatment-selection assay.

## 6 Challenges and limitations

The central translational challenge is the gap between statistical association and actionable clinical utility. A biomarker may be biologically plausible and independently associated with an outcome yet still fail to improve prediction, alter management or improve patient outcomes when added to established clinical assessment.

Despite the rapid development of research into CKM-related biomarkers, there remains a significant gap between the maturity of the evidence and clinical application needs. Most novel biomarkers are currently still in the discovery, association validation or risk prediction stages; research designs are predominantly cross-sectional studies, retrospective cohorts and secondary analyses of existing trials, and much of the evidence originates from single-disease populations such as those with diabetic kidney disease (DKD), CKD or heart failure, rather than from CKM cohorts defined according to uniform criteria (155, 208, 209). Consequently, the fact that a particular biomarker has an independent statistical association with an outcome does not equate to the conclusion that clinical measures based on that marker will improve patient outcomes. For example, recent studies have shown that natriuretic peptides or NT-proBNP can predict the risk of heart failure, cardiorenal events and mortality in patients with diabetes; however, the association between the biomarker and the outcome does not imply that interventions based on the test results will improve prognosis (210, 211). Future research will still need to conduct external validation and marker-guided intervention trials in CKM cohorts with standardised definitions to determine whether they can genuinely alter decisions regarding screening, referral, treatment and follow-up. Furthermore, for biomarkers to progress from correlational studies to clinical application, they must, at a minimum, meet the following criteria: test results must be stable and reproducible, validated in independent populations, and capable of providing incremental value beyond existing models. More importantly, test results should be capable of altering clinical decision-making and improving patient outcomes, while taking into account cost, accessibility and equity (212).

An additional limitation is the non-independence of the published evidence. Multiple studies of derived inflammatory, oxidative-stress and metabolic indices have reused overlapping national surveys or biobank populations. Reanalysis of the same source cohort with different indices or outcomes may increase the number of publications without providing truly independent replication. Future studies should report survey cycles, participant overlap and prior analyses transparently, and claims of reproducibility should be based on geographically and clinically independent external cohorts.

Insufficient standardisation of testing is also a significant issue affecting the clinical translation of novel biomarkers. For many biomarkers of inflammation, fibrosis, oxidative stress and omics, standardised procedures for sample collection and processing, cross-platform calibration systems, reference ranges and clinical decision thresholds have yet to be established (110, 213). Although composite indices such as eGDR, TyG, SII and SIRI are straightforward to calculate, differences between studies in terms of population composition, units of measurement and methods for determining cut-off points mean that the optimal thresholds obtained are not consistent (113). Omics test results are also susceptible to influences from age, sex, diet, medication, and sample processing and storage conditions (213). Furthermore, factors such as BMI, renal function and atrial fibrillation can alter the baseline levels of natriuretic peptides and other cardiovascular markers (11–15, 201, 206). Consequently, cut-off points derived from studies should not be directly applied to routine clinical decision-making until standardisation of testing, establishment of stratified reference intervals and external validation have been completed.

Discordant biomarker patterns are likely in CKM because production, tissue release, renal clearance and haemodynamic load can change simultaneously. For example, NT-proBNP may increase because of reduced kidney function but be relatively suppressed by obesity, while reduced eGFR may occur without albuminuria. Such discordance should not be resolved by automatically privileging a single marker. Clinicians should instead consider serial trajectories, complementary organ-specific information, pre-analytical factors and the overall clinical phenotype. Prospective studies are needed to determine whether predefined approaches to discordant biomarker profiles improve clinical decisions or outcomes.

A single biomarker cannot fully reflect the characteristics of CKM, which involves multiple organs and pathways simultaneously; nor does the value of combined testing depend solely on the number of markers. Some biomarkers have broad origins or are jointly influenced by multiple pathological processes, making it difficult to distinguish between metabolic abnormalities, inflammatory activation, haemodynamic load and structural organ damage, whereas other markers, although strongly indicative of specific mechanisms, still have limited sensitivity or specificity when used in isolation (107, 214). Combined models can integrate complementary information; however, an excessive number of markers may also increase multicollinearity, overfitting, missing data, testing costs and the difficulty of interpreting results (107, 168). Therefore, models should be constructed around a clear clinical purpose and evaluated against traditional risk models for incremental predictive value, calibration, external validity, clinical net benefit and cost-effectiveness. Dynamic monitoring may better reflect disease progression and treatment response than a single test; however, the clinical significance of any changes should be assessed in the context of analytical error, intra-individual biological variation, reference change values and short-term physiological fluctuations (215).

Population differences may also affect the generalisability of research findings. Age, sex, ethnicity, degree of obesity, CKD stage, comorbidities and treatment status may all alter the distribution and predictive ability of biomarkers. Some models are based primarily on populations of European descent, while research findings in Asian diabetic populations require validation in other regions (115, 175, 211). Future research should be conducted in multicentre, multiethnic populations with varying healthcare settings, and the accuracy, equity and clinical value of the models should be continuously evaluated and calibrated across different subgroups.

In addition to biological heterogeneity, testing costs, reimbursement coverage and access to services remain important barriers to the clinical translation of CKM biomarkers. The AHA has emphasised that populations disadvantaged by adverse social determinants of health bear a disproportionate burden of CKM; evaluation of new biomarker technologies should therefore include health-equity considerations (7). Routine measures such as serum creatinine-based eGFR, UACR, blood glucose and lipid profiles can generally be incorporated into existing primary-care pathways. A Chinese modelling study suggested that population-based CKD screening using eGFR and UACR may be cost-effective under defined assumptions; however, these findings depend on successful diagnostic confirmation, treatment uptake and long-term adherence and cannot be extrapolated directly to novel biomarker or multi-omics testing (216). Emerging kidney biomarkers and multi-omics assays may require specialised analytical platforms, standardised pre-analytical procedures and additional quality control, while evidence regarding their incremental clinical utility, implementation costs and cost-effectiveness remains limited (213, 217). In its assessment of NGAL- and cell-cycle-arrest-based tests for acute kidney injury, National Institute for Health and Care Excellence (NICE) found no evidence that test-guided preventive care improved clinical outcomes and considered the available cost-effectiveness estimates highly uncertain (218). Reimbursement is also context-specific. For example, a US Medicare Administrative Contractor local coverage determination provides conditional, once-in-a-lifetime coverage of KidneyIntelX or KidneyIntelX.dkd for selected adults with type 2 diabetes and stage 1–3b CKD, rather than establishing universal national coverage (219). Future studies should therefore report not only predictive performance but also assay and downstream management costs, budget impact, out-of-pocket expenditure, test completion, clinical net benefit and cost-effectiveness across socioeconomic and healthcare-access groups. High-cost testing should not become a prerequisite for evidence-based CKM treatment or widen existing health inequalities.

## 7 Future directions and prospects for clinical application

Future research should focus on technology integration, mechanism validation, clinical translation and cross-disciplinary collaboration, with an emphasis on determining whether new biomarkers can provide reproducible, incremental information that can be translated into actionable and equitable management strategies.

### 7.1 Technology integration

A stratified, modular CKM biomarker system should align test intensity with the clinical question and evidence tier: the baseline layer would utilise blood pressure, HbA1c, lipid profile, BMI, eGFR and UACR; the organ damage layer would incorporate NT-proBNP, hs-cTn and indicators of tubular damage; and the mechanism layer would further integrate data on inflammation, fibrosis, oxidative stress, metabolomics, imaging and digital data (155). Testing intensity should increase with clinical risk and be tailored to the clinical question, and any new modules must demonstrate stable incremental value relative to existing models (175, 185, 212). Artificial intelligence can integrate high-dimensional and longitudinal data; however, in addition to area under the curve (AUC), model development should also report on external validation, calibration, interpretability, cross-population transferability and subgroup equity, and be linked to explicit action rules within electronic health records (168).

### 7.2 In-depth mechanistic studies

Mechanistic studies should distinguish between risk signals, causal mediators and druggable targets. For sTNFR1/2, suPAR, GDF-15, sST2, galectin-3 and collagen turnover markers, longitudinal measurements, genetic causal analysis, tissue expression and intervention studies can be combined to examine whether changes in these markers correlate with clinical benefit (24, 104, 136, 138, 140, 197, 209, 220). Single-cell and spatial multi-omics, extracellular vesicles, patient-derived cells and multi-organ chips can be utilised to elucidate and validate causal pathways between the heart, kidneys, adipose tissue and blood vessels (155, 162–166, 221, 222). The focus of research should shift from identifying additional relevant molecules to determining key nodes that are reproducible, amenable to intervention and capable of improving outcomes.

### 7.3 Translational research

Clinical application should adopt a tiered screening strategy that balances resource availability with health equity. Primary care systems should ensure that populations with indications for screening—particularly people in rural areas and low-income groups—have access to essential tests such as blood pressure, BMI, HbA1c, lipid profile, creatinine/eGFR, UACR and serum potassium at a low cost. For high-risk individuals, those with rapid disease progression or those with an unclear clinical phenotype, further testing for organ damage markers such as NT-proBNP and hs-cTn may be conducted at regional medical centres. Tests for KIM-1, NGAL, sTNFR1/2, fibrosis markers and multi-omics analyses should be reserved for specific scenarios where the results may influence decisions regarding referral, treatment or follow-up; barriers to their use can be reduced through centralised testing, specimen transport and conditional coverage (7, 218, 219). It should be emphasised that the unavailability of high-cost tests should not prevent patients from receiving treatments with established indications based on clinical phenotype and routine indicators such as eGFR and UACR. Evaluations of tiered strategies should compare overall cost-effectiveness, test completion rates, misdiagnosis rates, referral rates, out-of-pocket costs and outcome benefits across populations with different income levels, health insurance types, urban/rural settings and levels of healthcare resources, to ensure that new technologies narrow rather than widen health disparities in CKM.

### 7.4 Cross-disciplinary collaboration

Progress in CKM is not only linked to biological mechanisms such as inflammation and metabolic disorders, but is also influenced by sleep, psychological state, physical activity and the social environment. A UK Biobank study involving 375,837 participants found that individuals with better sleep quality had a lower risk of developing their first cardiovascular, renal or metabolic disease, as well as a lower risk of further progression to multimorbidity (hazard ratio [HR] = 0.70 for both). Anxiety or depression may account for 16.48% of the association between sleep and disease onset (186). This suggests that sleep and psychological state may provide information beyond that offered by traditional biomarkers.

In future, data on sleep, physical activity and heart rate recorded by wearable devices could be combined with psychological assessments, social environment data and laboratory indicators to form a comprehensive ‘molecular-behavioural-psychological-social’ management model (7, 8, 223, 224). For identified modifiable risks, interventions such as sleep improvement, psychological support, exercise and nutritional guidance could be tailored to individual circumstances, with effects evaluated through multidisciplinary collaboration and regular monitoring (225).

Overall, the focus of CKM biomarker research should not be merely on increasing the number of markers, but rather on identifying stable, reproducible markers that offer incremental clinical value. Through standardised testing, multicentre validation, the integration of artificial intelligence and multidisciplinary collaboration, a dynamic management system that takes into account biological mechanisms, clinical phenotypes and socio-behavioural factors should be established to advance the early identification of CKM and personalised interventions.

## 8 Conclusion

CKM biomarkers should be interpreted according to their level of evidence and clinical maturity. Established biomarkers, including eGFR, UACR, BNP/NT-proBNP and hs-cTn, remain the foundation of screening, staging, risk assessment and safety monitoring across CKM component conditions. Selected adjunctive tests, such as cystatin C when creatinine-based eGFR may be unreliable, can refine assessment in defined clinical contexts. In contrast, most inflammatory, oxidative-stress, tubular-injury, fibrosis, derived composite, non-coding RNA, extracellular-vesicle and multi-omics markers remain investigational. Although many are biologically plausible and prognostically associated with outcomes, few have demonstrated consistent incremental value, external validity, actionable thresholds or improved outcomes in biomarker-guided intervention trials.

Routine adoption of emerging biomarkers should therefore require analytical standardisation, prospective external validation, demonstration of incremental clinical net benefit and, where relevant, biomarker-guided trials showing that their use changes treatment decisions and improves patient outcomes. The most clinically useful future strategy is likely to combine parsimonious biomarker panels with longitudinal clinical data, imaging and digital measures while preserving interpretability, feasibility and equity. Until these requirements are met, emerging biomarkers should complement rather than replace established clinical assessment.

## Glossary

AAA: Aromatic amino acid
ACC: American College of Cardiology
ACEI: Angiotensin-converting enzyme inhibitor
ADA: American Diabetes Association
ADMA: Asymmetric dimethylarginine
AGR: Albumin-to-globulin ratio
AHA: American Heart Association
AI: Artificial intelligence
AIP: Atherogenic index of plasma
AKI: Acute kidney injury
ANLR: Albumin-to-neutrophil/lymphocyte ratio
AOPP: Advanced oxidation protein products
ARB: Angiotensin receptor blocker
ASCVD: Atherosclerotic cardiovascular disease
ASN: American Society of Nephrology
AUC: Area under the curve
BCAA: Branched-chain amino acid
bio-ADM: Biologically active adrenomedullin
BMI: Body mass index
BNP: B-type natriuretic peptide
CA125: Carbohydrate antigen 125
CAT: Catalase
CDAI: Composite Dietary Antioxidant Index
CHARLS: China Health and Retirement Longitudinal Study
CKD: Chronic kidney disease
CKM: Cardiovascular-kidney-metabolic syndrome
CMS: Centers for Medicare &
CRIC: Chronic Renal Insufficiency Cohort
CRP: C-reactive protein
CTGF: Connective tissue growth factor
CVAI: Chinese visceral adiposity index
CVD: Cardiovascular disease
DALYs: Disability-adjusted life years
DII: Dietary Inflammatory Index
DKD: Diabetic kidney disease
E-DII: Energy-adjusted Dietary Inflammatory Index
eGDR: Estimated glucose disposal rate
eGFR: Estimated glomerular filtration rate
eGFRcr-cys: Estimated glomerular filtration rate based on creatinine and cystatin C
ET-1: Endothelin-1
EV: Extracellular vesicle
FABP4: Fatty acid-binding protein 4
FGF-23: Fibroblast growth factor 23
GDF-15: Growth differentiation factor 15
GFR: Glomerular filtration rate
GLP-1 RA: Glucagon-like peptide-1 receptor agonist
GPx: Glutathione peroxidase
HbA1c: Glycated haemoglobin
HDL-C: High-density lipoprotein cholesterol
H-FABP: Heart-type fatty acid-binding protein
HOMA-IR: Homeostatic model assessment for insulin resistance
hs-CRP: High-sensitivity C-reactive protein
hs-cTn: High-sensitivity cardiac troponin
hs-cTnI: High-sensitivity cardiac troponin I
hs-cTnT: High-sensitivity cardiac troponin T
IDI: Integrated discrimination improvement
IGFBP7: Insulin-like growth factor-binding protein 7
IL: Interleukin
IR: Insulin resistance
IS: Indoxyl sulphate
KDIGO: Kidney Disease, Improving Global Outcomes
KIM-1: Kidney injury molecule-1
LAP: Lipid accumulation product
L-FABP: Liver-type fatty acid-binding protein
lncRNA: Long non-coding RNA
LVEF: Left ventricular ejection fraction
MASLD: Metabolic dysfunction-associated steatotic liver disease
MDA: Malondialdehyde
METS-IR: Metabolic score for insulin resistance
miRNA: MicroRNA
ML: Machine learning
MMP: Matrix metalloproteinase
MRA: Mineralocorticoid receptor antagonist
MR-proADM: Mid-regional pro-adrenomedullin
NAG: N-acetyl-β-D-glucosaminidase
NGAL: Neutrophil gelatinase-associated lipocalin
NHANES: National Health and Nutrition Examination Survey
NHHR: Non-high-density lipoprotein cholesterol-to-high-density lipoprotein cholesterol ratio
NHR: Neutrophil-to-high-density lipoprotein cholesterol ratio
NICE: National Institute for Health and Care Excellence
NLR: Neutrophil-to-lymphocyte ratio
NO: Nitric oxide
NRI: Net reclassification improvement
NT-proBNP: N-terminal pro-B-type natriuretic peptide
OBS: Oxidative Balance Score
ox-LDL: Oxidised low-density lipoprotein
PAI-1: Plasminogen activator inhibitor-1
PCS: p-Cresyl sulphate
PLR: Platelet-to-lymphocyte ratio
PREVENT: Predicting Risk of Cardiovascular Disease EVENTs
RAAS: Renin–angiotensin–aldosterone system
RC: Remnant cholesterol
RCT: Randomised controlled trial
ROS: Reactive oxygen species
SGLT2i: Sodium–glucose cotransporter-2 inhibitor
SII: Systemic immune-inflammation index
SIRI: Systemic inflammation response index
SOD: Superoxide dismutase
sST2: Soluble suppression of tumorigenicity 2
sTNFR1/2: Soluble tumour necrosis factor receptors 1 and 2
suPAR: Soluble urokinase-type plasminogen activator receptor
T2DM: Type 2 diabetes mellitus
TAC: Total antioxidant capacity
TIMP-2: Tissue inhibitor of metalloproteinases 2
TMAO: Trimethylamine N-oxide
TMAVA: N,N,N-Trimethyl-5-aminovaleric acid
TNF-α: Tumour necrosis factor-α
TyG: Triglyceride-glucose index
TyG-WC: Triglyceride-glucose–waist circumference index
UACR: Urine albumin-to-creatinine ratio
VAI: Visceral adiposity index
vWF: von Willebrand factor
WHtR: Waist-to-height ratio
4-HNE: 4-Hydroxynonenal
8-OHdG: 8-Hydroxy-2′-deoxyguanosine.
